# Placentation in Sigmodontinae: a rodent taxon native to South America

**DOI:** 10.1186/1477-7827-9-55

**Published:** 2011-04-25

**Authors:** Phelipe O Favaron, Anthony M Carter, Carlos E Ambrósio, Adriana C Morini, Andrea M Mess, Moacir F de Oliveira, Maria A Miglino

**Affiliations:** 1 Department of Surgery, School of Veterinary Medicine, University of Sao Paulo, Sao Paulo, Brazil; 2 Cardiovascular and Renal Research, Institute of Molecular Medicine, University of Southern Denmark, Odense, Denmark; 3 Department of Basic Science, Faculty of Animal Sciences and Food Engineering, University of São Paulo, Pirassununga, Brazil; 4 Department of Animal Science, Universidade Federal Rural do Semi-Árido, Mossoró, Rio Grande do Norte, Brazil

## Abstract

**Background:**

Sigmodontinae, known as "New World rats and mice," is a large subfamily of Cricetidae for which we herein provide the first comprehensive investigation of the placenta.

**Methods:**

Placentas of various gestational ages ranging from early pregnancy to near term were obtained for five genera, i.e. *Necromys, Euryoryzomys, Cerradomys, Hylaeamys*, and *Oligoryzomys*. They were investigated by means of histology, immunohistochemistry, a proliferation marker, DBA-lectin staining and transmission electron microscopy.

**Results:**

The chorioallantoic placenta was organized in a labyrinthine zone, spongy zone and decidua and an inverted yolk sac persisted until term. The chorioallantoic placenta was hemotrichorial. The interhemal barrier comprised fetal capillary endothelium and three layers of trophoblast, an outermost, cellular layer and two syncytial ones, with interspersed trophoblast giant cells (TGC). In addition, accumulations of TGC occurred below Reichert's membrane. The junctional zone contained syncytial trophoblast, proliferative cellular trophoblast, glycogen cells and TGC that were situated near to the maternal blood channels. In three of the genera, TGC were also accumulated in distinct areas at the placental periphery. PAS-positive glycogen cells derived from the junctional zone invaded the decidua. Abundant maternal uNK cells with positive response to PAS, vimentin and DBA-lectin were found in the decidua. The visceral yolk sac was completely inverted and villous.

**Conclusion:**

The general aspect of the fetal membranes in Sigmodontinae resembled that found in other cricetid rodents. Compared to murid rodents there were larger numbers of giant cells and in some genera these were seen to congregate at the periphery of the placental disk. Glycogen cells were found to invade the decidua but we did not identify trophoblast in the walls of the deeper decidual arteries. In contrast these vessels were surrounded by large numbers of uNK cells. This survey of wild-trapped specimens from five genera is a useful starting point for the study of placentation in an important subfamily of South American rodents. We note, however, that some of these rodents can be captive bred and recommend that future studies focus on the study of time dated pregnancies.

## Background

Muridae and Cricetidae are the most species-rich families of rodents, with around 600 species in each family [1]. For both taxa there are still important gaps in basic knowledge about reproductive biology. In particular, the development of the placenta is not well understood for the majority of species. Muridae includes several laboratory models, such as mouse and rat, for which placentation is very well documented [e.g., 2-9], yet 98% of the murid species have not been studied with regard to their placentation [10]. Cricetidae is even less well covered although some basic data is available for the golden hamster *Mesocricetus auratus *from the subfamily Cricetinae [11-13]. In addition, some aspects of placentation have been documented for members of the subfamily Arvicolinae, namely voles and lemmings [14,15], and the North American deer mouse *Peromyscus maniculatus *from the subfamily Neotominae [15,16]. But for the largest subfamily, the Sigmodontinae, with 377 recognized species in 74 genera [1], data on placentation is very sparse. The only species studied so far is *Calomys callosus*, which has been used as an experimental model for parasitological and other diseases [17-22].

Sigmodontine rodents form a monophyletic clade [23-25]. These rodents are largely confined to the Neotropics [26], and are often referred to as "New World rats and mice". They are known to transmit diseases to humans and domestic animals. Antibody prevalence indicates that sigmodontine species are reservoirs of Hantavirus in the several regions of Brazil and other parts of Latin America [27]. In addition, they are the most important reservoirs of zoonotic cutaneous leishmaniasis throughout their range [24]. For this reason they are generally considered as pests. On the other hand, sigmodontine rodents usually possess restricted ranges, and are vulnerable to habitat loss. Thus, they may serve as indicators for biodiversity purposes [28]. A better understanding of reproductive biology in these species is therefore desirable and needs to include a comprehensive analysis of placental development and structure.

We here provide a detailed study on placentation in five genera of sigmodontine rodents (*Necromys, Euryoryzomys, Cerradomys, Hylaeamys *and *Oligoryzomys*). Data on reproduction in this subfamily is sparse but where known gestation lasts 23 to 30 days, slightly longer than in mice, rats and other cricetids [9,12,26,29]. Placentas from various gestational stages, ranging from early pregnancy to near term, have been studied by a variety of techniques including immunohistochemistry, DBA-lectin staining and transmission electron microscopy. The findings are compared and contrasted with what is known about placentation in other murid and cricetid rodents.

## Methods

### Material

Placentae from five genera of sigmodontine rodents were investigated: *Necromys, Euryoryzomys, Cerradomys, Hylaeamys *and *Oligoryzomys *(Table 1). The nomenclature follows a recent revision of the *Oryzomys *complex by Weksler et al. [25]. Most of the material was obtained from the collection of the Museum of Zoology, University of Sao Paulo (MZUSP). Additional material of *Necromys *was obtained from a breeding group at the Universidade Federal Rural do Semi Árido, Mossoró, Rio Grande do Norte (CEMAS). *Euryoryzomys *was live-trapped at Sao Joaquim da Barra, Sao Paulo (SJB). The identification of *Euryoryzomys *was made by the Laboratory of Ecology and Evolution, Butantan-Institute, Sao Paulo. Voucher material was given to the Museum of Veterinary Anatomy (MAV), University of Sao Paulo. The other material is now housed at the School of Veterinary Medicine, University of Sao Paulo.

**Table 1:** Material collected with values

		Fetuses		Placentae			
Species and Collection Number	Geographical Origin	FL (cm)	FW (g)	PW (g)	PV (ml)	PL (cm)	PD (cm)
***Necromys lasiurus***							
MAV (CEMAS 03)	Mossoro, RN	Implantation	-	-	-	-	-
MAV (CEMAS 04)	Mossoro, RN	0.5	0.148	0.052	0.2	0.5	0.3
MAV (CEMAS 05)	Mossoro, RN	0.6	0.151	0.065	0.2	0.6	0.4
MZUSP (APC 1140)	Santa Bárbara, SP	1.3	0.377	0.142	0.4	1.1	0.9
MZUSP (APC 1246-1)	Serra Geraldo Tocantins, TO	3.1	3.354	0.696	0.9	1.4	0.7
MZUSP (APC 1246-2)	Serra Geraldo Tocantins, TO	3.1	3.432	0.407	0.5	1.3	1.1
MZUSP (APC 1246-3)	Serra Geraldo Tocantins, TO	2.5	1.964	0.324	0.5	1.2	0.6
MAV (CEMAS 01)	Mossoro, RN	near term	-	-	-	-	-
MAV (CEMAS 02)	Mossoro, RN	near term	-	-	-	-	-
***Cerradomys *gr. *subflavus***							
MZUSP (APC 1157)	Santa Barbara, SP	2.8	5.223	0.627	0.9	1.5	1.2
MZUSP (APC 1177)	Santa Barbara, SP	2.0	0.834	0.897	1.0	1.2	0.9
MZUSP (APC 1177-1)	Santa Barbara, SP	2.4	0.329	0.334	0.5	1.1	0.8
MZUSP (APC 1177-2)	Santa Barbara, SP	2.2	0.312	0.299	0.5	1.0	0.7
***Euryoryzomys *sp**.							
MAV (SJB 01)	São Joaquim da Barra, SP	1.6	0.907	-	-	-	-
MAV (SJB 02)	São Joaquim da Barra, SP	1.8	0.933	-	-	-	-
***Hylaeamys megacephalus***							
MZUSP (APC 1022-1)	Serra das Araras, MT	-	-	0.222	0.1	1.0	0.5
MZUSP (APC 1022-3)	Serra das Araras, MT	-	-	0.095	0.1	1.0	0.6
MZUSP (MRT 08415)	Goiania, GO	1	0.107	0.209	0.4	0.9	0.6
MZUSP (MRT 08408)	Goiania, GO	2.2	1.082	0.538	0.9	1.1	1.0
***Oligoryzomys *sp**.							
MZUSP 32729-1	Cotia and Ibiuna, SP	2.4	1.186	0.079	0.2	1.2	0.8
MZUSP 32729-4	Cotia and Ibiúna, SP	2.3	1.188	0.174	0.3	1.1	0.8
MZUSP 31167	Piedade, SP	1.0	0.118	0.051	0.1	0.6	0.5
MZUSP 32735	Piedade, SP	0.5	0.025	0.075	0.1	0.6	0.5

Fetal length (FL), fetal weight (FW), placental weight (PW), placental volume (PV), placental length (PL) and placental diameter (PD). Most specimens were from the collection of the Museum of Zoology, University of Sao Paulo, SP, Brazil (MZUSP) with temporary museum numbers given in brackets. The nomenclature follows a recent revision of *Oryzomys *by Weksler et al. [25]. Fresh material was collected at CEMAS, Mossoro, RN, and in São Joaquim da Barra (SJP), SP.

The museum specimens had been fixed in formaldehyde and stored in 70% alcohol. Tissue was processed by standard methods, embedded in paraffin (Paraplast; Oxford Labware, St Louis, MO, USA) and sectioned at 5 μm using an automatic microtome (Leica, RM2155, Germany).

### Histology, immunohistochemistry and lectin staining

Sections were stained with hematoxylin and eosin (HE), Masson's trichrome, picrosirius, and periodic acid-Schiff (PAS). In addition, immunohistochemistry was performed following the approaches used in previous studies from our laboratory [see 30,31]. Cytokeratin was used to identify trophoblast cells and was detected by a rabbit polyclonal antibody (1:300; PU071-UP, Biogenex, San Ramon, California, U.S.A.). Mouse monoclonal anti-human primary antibodies were used to detect vimentin (1:300; V9, sc-6260, Santa Cruz Biotechnology, Santa Cruz, California, USA), α-smooth muscle actin (1:300; Clone 1A4, DakoCytomation, Carpinteria, California, USA), and PCNA (1:400; PC10, sc-56, Santa Cruz Biotechnology, Santa Cruz, California, USA). Negative controls were performed using anti-mouse IgG (1:500; AP308F, Chemicon International Temecula, California, USA) as the primary antibody solution.

In addition, samples from three species (*Necromys lasiurus, Hylaeamys megacephalus*, and *Cerradomys *gr. *subflavus*,) were stained using a *Dolichos biflorus *(DBA) lectin to identify mature uNK cells, following the protocol described by Zhang et al. [4].

### Transmission electron microscopy

Samples for transmission electron microscopy, derived from freshly obtained material of *Necromys *and *Euryoryzomys*, were fixed in 2.5% glutaraldehyde. Tissues were maintained in this solution for 48 h and post-fixed for 2 h in 2% phosphate-buffered osmium tetroxide (pH 7.4, for 2 h), then washed in phosphate buffer (3 × 10 min) and immersed in 3% uranyl acetate solution overnight. After being re-washed in buffer (3 × 10 min), tissues were dehydrated in alcohol and immersed in propylene oxide for 10 min. Finally, the samples were embedded in Spurr's Resin (Polysciences, Warrington, PA, USA). Ultrathin sections were made on an automatic ultramicrotome (Ultracut R, Leica Microsystems, Germany), contrasted with 2% uranyl acetate and 0.5% lead citrate and studied in a transmission electron microscope (Morgagni 268D, FEI Company, The Netherlands; Mega View III camera, Soft Imaging System, Germany).

## Results

### Macroscopic structure

**Figure 1:**
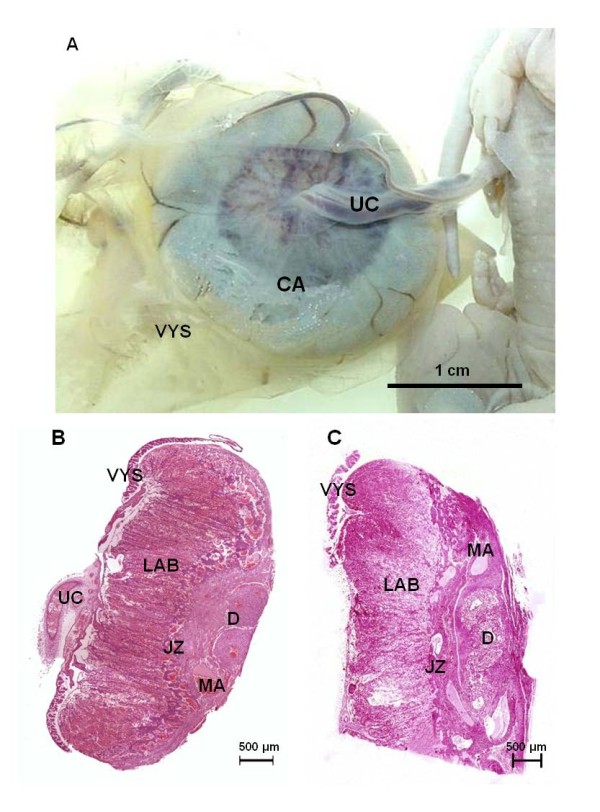
**Gross morphology**. **(A) ***Cerradomys*, near term (MZUSP/APC-1157). Chorioallantoic placenta (CA), umbilical cord (UC) and visceral yolk sac (VYS). **(B) **Histology of the placenta near term in *Cerradomys *(MZUSP/APC 1177-1) with the main regions of interest including decidua (D) with supplying maternal arteries (MA), junctional zone (JZ), and labyrinth (LAB). **(C) ***Oligoryzomys*. Near term placenta (MZUSP 32729-1).

In all taxa investigated the chorioallantoic placenta was situated at the antimesometrial side of the bicornuate uterus and a choriovitelline placenta persisted throughout gestation (Figures 1A-C). The embryos were situated towards the mesometrial side of the uterus. The definitive chorioallantoic placenta was discoidal to ovoid in shape. The junction between the chorion and the uterus was restricted to an area at the antimesometrial side of the uterus (Figures 1B,C). A delicate umbilical cord was attached to the center of the placental disk. The visceral yolk sac was completely inverted at all stages (Figures 1B,C).

### Labyrinth

**Figure 2:**
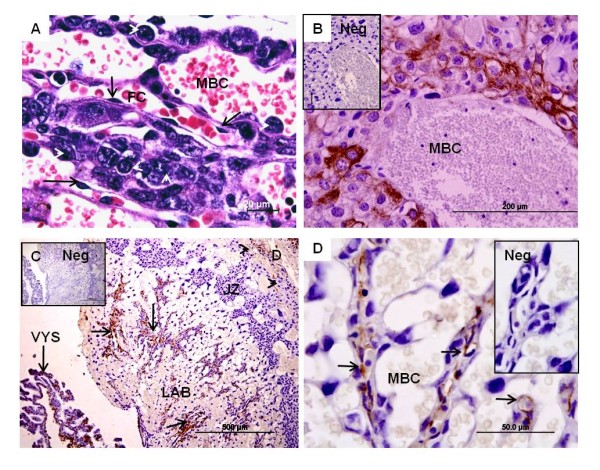
**Main regions of the chorioallantoic placenta**. **(A) ***Cerradomys*, near term (MZUSP/APC 1177-1). HE. Trophoblast layers separated maternal blood channels (MBC) and fetal capillaries in the labyrinth; the latter with intact endothelium (arrows). The placental barrier varied, possessing very thin areas and some thicker regions that included sinusoid trophoblast giant cells (arrowheads). **(B) ***Necromys*, near term (MAV/CEMAS 01). Immunohistochemistry for cytokeratin. In contrast to the labyrinth, the junctional zone did not possess fetal vessels. The maternal blood channels in this area were associated with trophoblast. **(C) ***Necromys *in mid gestation (MZUSP/APC 1140). Immunohistochemistry for vimentin showed positive response for the fetal blood vessels (arrow) in the labyrinth (LAB), indicating an intact endothelium. In the visceral yolk sac (VYS) mesoderm was stained. In contrast, the maternal blood system of the labyrinth and junctional zone (JZ) lack endothelium, resulting in the vimentin-negative response of the junctional zone. In the decidua (D), maternal cells of mesenchymal origin were stained (arrow heads). **(D) ***Necromys *in mid gestation (MZUSP/APC 1140). Higher magnification of the labyrinth. Fetal vessels (arrow) ran inside the trophoblastic (vimentin-negative) layers that lined the maternal blood channels.

The chorioallantoic placenta was composed of a labyrinth situated towards the fetal or the mesometrial side of the uterus and an inner junctional zone (or trophospongium) adjacent to the decidua (Figures 1B,C). The labyrinth was vascularized from both the maternal and fetal circulations. The maternal blood was found to flow through tubular channels, the maternal blood lacunae or blood channels (Figure 2A), also found in the junctional zone (Figure 2B). The fetal vessels had intact endothelium, resulting in vimentin-positive response of the labyrinth (Figures 2C,D). The walls of the lacunae were composed of syncytial and cellular trophoblast. In addition to normal-sized trophoblasts, larger cells were interspersed, the sinusoidal trophoblast giant cells (Figure 2A). Thus, the barrier varied in thickness, including thinner and thicker regions. In addition, groups of TGC occurred at the outer border of the labyrinth, adjacent to Reichert's membrane.

### Interhemal barrier

**Figure 3:**
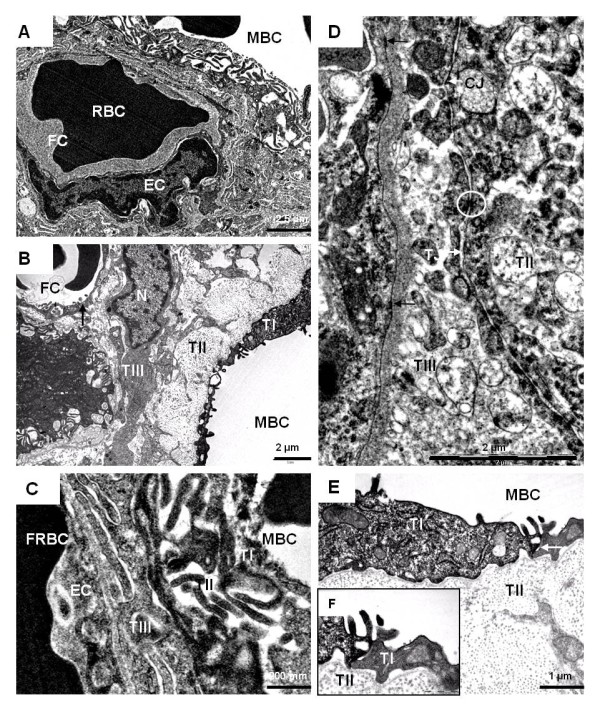
**Ultrastructure of the labyrinth in mid gestation**. **(A) ***Necromys *(MZUSP/APC 1246-3). The placental barrier separated a maternal blood channel (MBC) and a fetal capillary (FC) with intact endothelium (EC) and a red blood cell (RBC). **(B) ***Necromys *(MZUSP/APC 1246-3). The placenta comprised three trophoblastic layers (TI, TII and TIII). **(C) ***Euryoryzomys *(MAV/SJB 02). The hemotrichorial placenta. **(D) ***Necromys *(MZUSP/APC 1246-3). There was a strong contact between layer TIII and the basement membrane of the endothelial cell (arrow). Different types of junctions occurred between trophoblast layers TII and TIII, including tight junctions (TJ), intermediate junctions (CJ) and desmosomes (circle). **(E) ***Necromys *(MZUSP/APC 1246-3). The outermost layers in detail. **(F) ***Euryoryzomys *(MAV/SJB 02). Two cells belonging to layer TI.

The labyrinth is the region of feto-maternal exchange. Trophoblastic trabeculae formed the barrier between the fetal and maternal circulations. In contrast to the fetal system that had intact capillaries, the maternal blood flowed through trophoblast-lined channels (Figures 3A-C). As mentioned above, the thickness of the placental barrier varied greatly in all taxa investigated, including very thin areas but also thicker parts (Figure 2A). However, the minimal thickness in the two taxa that were studied by electron microscopy was found to be approximately 2.5 μm in *Necromys *and 7 μm in *Euryoryzomys*. The placental barrier comprised three trophoblastic layers (Figures 3B-F), resulting in a hemotrichorial condition. The outermost layer (TI) lined the maternal blood channels. It was cellular in nature (Figures 3E,F). In places this layer (TI) was reduced to a very thin flange, but with projections towards the maternal blood (Figures 3E,F). The middle layer (TII) was much thicker and only loosely attached to the layer above it. It was syncytial in nature (not shown). However, an irregularly folded structure was present. Finally, the innermost layer (TIII) was likewise syncytial (Figures 3B,C). It was very closely attached to the neighboring layer (TII), including tight and intermediate junctions as well as desmosomes (Figure 3D). Moreover, this layer (TIII) had a strong connection to the basement membrane of the fetal capillary endothelium (Figure 3D). In the areas of the labyrinth that were characterized by a thicker barrier between the two circulations, additional trophoblast and sinusoidal trophoblast giant cells were present below the innermost layer (TIII). It was mostly cellular.

### Junctional zone and giant cell region

**Figure 4:**
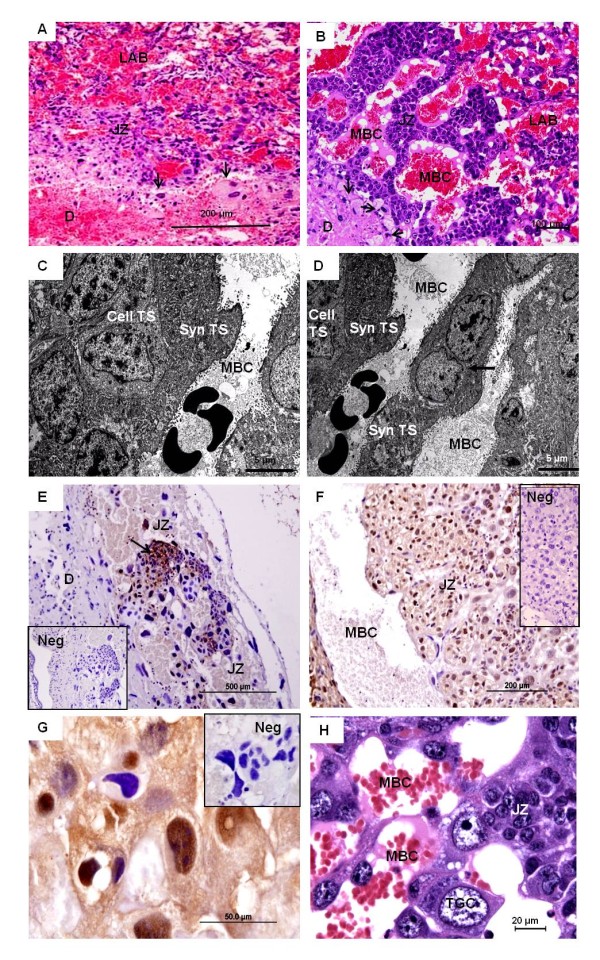
**The junctional zone**. **(A) ***Necromys*, early pregnancy (MAV/CEMAS 05). HE. The junctional zone (JZ) with simple structure facing towards the decidua. The border was not sharp with trophoblast derivatives (arrows) invading into the decidua. **(B) ***Cerradomys*, near term (MZUSP/APC 1157). The junctional zone had a folded structure arranged around the maternal blood channels (MBC). **(C) ***Necromys*, near term (MAV/CEMAS 02). TEM. Syncytial trophoblast (Syn TS) lined the maternal blood channels (MBC), associated with underlying cellular trophoblast (Cell TS). **(D) ***Necromys*, near term (MAV/CEMAS 02). TEM. In places the trophoblast separating the maternal blood spaces was represented only by a thin syncytial layer (arrow). **(E) ***Necromys *in early pregnancy (MAV/CEMAS 05). PCNA. Clustered groups of proliferating trophoblast cells (arrow) occurred in the junctional zone. **(F) ***Necromys *in mid gestation (MZUSP/APC 1246-1). PCNA. In more advanced stages, proliferating cells were widespread in the junctional zone. **(G) ***Necromys *in mid gestation (MZUSP/APC 1246-1). PCNA. Higher magnification. **(H) ***Cerradomys*, near term (MZUSP/APC 1157). HE. Among the spongiotrophoblast in the junctional zone, trophoblast giant cells (TGC) occurred. They were close to the maternal blood spaces and had large nuclei and prominent chromatin.

**Figure 5:**
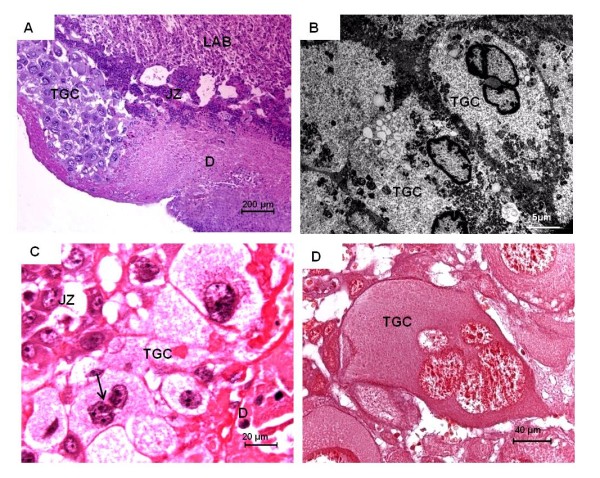
**Giant cells**. **(A) ***Euryoryzomys *in mid gestation (MAV/SJB 01). HE. Trophoblast giant cells (TGC) were accumulated at the outer areas of the placental disk, adjacent to the labyrinth (LAB) and junctional zone (JZ). **(B) ***Euryoryzomys *in mid gestation (MAV/SJB 02). TEM. Trophoblast giant cells at higher magnification. These cells were binucleate in nature. **(C) ***Cerradomys*, near term (MZUSP/APC 1177-1). HE. A continuous layer of trophoblast giant cells (TGC) was situated between the junctional zone (JZ) and the decidua (D). Some cells were binucleate (arrow). **(D) ***Oligoryzomys*, near term (MZUSP 32729-1). HE. Some trophoblast giant cells were multinucleate.

The junctional zone (Figures 4A,B) abutted the decidual region. It consisted largely of spongiotrophoblast. In most areas, both syncytial and cellular trophoblast were found (Figure 4C), but in some places only a thin syncytial layer was associated with the maternal blood spaces (Figure 4D), whereas in others there were trophoblast cells. The trophoblast cells were actively proliferative, as indicated by PCNA-staining. In early pregnancy, such proliferative trophoblast clustered as nests inside the junctional zone (Figure 4E), whereas it was more frequent and distributed throughout this region in more advanced pregnancies (Figures 4F,G). Moreover, trophoblast giant cells were dispersed within the junctional zone, closely associated with the maternal blood channels (Figures 4H). They had large nuclei and prominent chromatin. The largest amount of trophoblast giant cells inside the junctional zone was found in *Oligoryzomys *and *Necromys*, in both closely associated with the spongiotrophoblast. In the three other genera of sigmodontine rodents that had fewer trophoblast giant cells inside the junctional zone - *Cerradomys, Hylaeamys *and *Euryoryzomys *- trophoblast giant cells were found to be accumulated in distinct regions at the periphery of the placental disk (Figures 5A,B). All investigated genera of sigmodontine rodents possessed a continuous band of trophoblast giant cells, situated between the decidua and the junctional zone (Figure 5C). This band was connected to the region of accumulated trophoblast giant cells in the three above mentioned genera. The giant cells were mostly bi- or multinucleated in nature.

**Figure 6:**
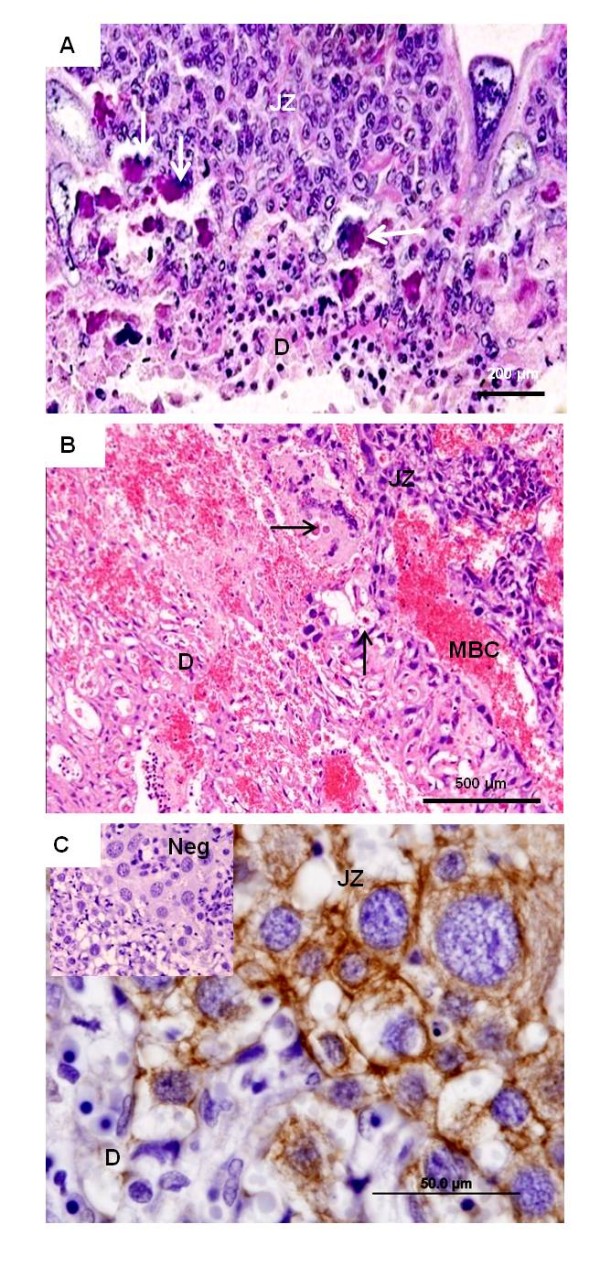
**Glycogen cells in mid gestation**. **(A) ***Hylaeamys *(MZUSP/APC 1022-1). PAS. Glycogen cells (arrows) at the outer margin of the junctional zone (JZ) just above the decidua (D). **(B) ***Necromys *(MZUSP/APC 1140). HE. Glycogen cells (arrows) invading the decidua near a maternal blood channel (MBC) that was entering the placenta. **(C) ***Necromys *(MZUSP/APC 1140). Immunohistochemistry for cytokeratin confirmed the trophoblastic nature of cells that originated from the junctional zone and invaded the decidua.

Neither the spongiotrophoblast nor the trophoblast giant cells associated with the junctional zone showed positive reactions to PAS staining. This distinguished them from another type of cell, the so-called glycogen cells, the only type of fetal cell that showed positive reaction to PAS (Figure 6A). The glycogen cells were present inside the junctional zone as well as on the outer border, from which they invaded into the decidual region (Figures 6A-B). This extraplacental trophoblast was identified by cytokeratin-immunopositivity (Figure 6C), and was vimentin-negative.

### Decidua and maternal blood supply

**Figure 7:**
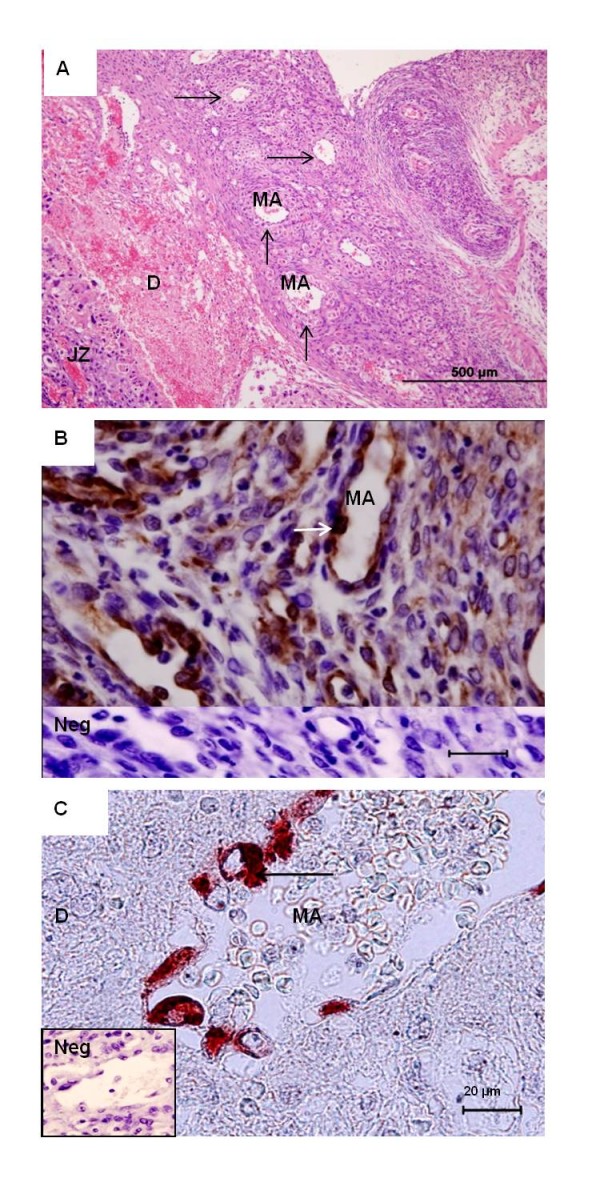
**Decidua**. **(A) ***Necromys *in mid gestation (MZUSP/APC 1140). HE. Maternal spiral arteries (MA) in the decidua (D) on their way to the junctional zone (JZ). They were associated with large cells (arrows). **(B) ***Necromys*, near term (MAV/CEMAS 01). Immunohistochemistry for vimentin. An artery in the mesometrium possessed an intact endothelium (arrow). **(C) ***Oligoryzomys*, near term (MZUSP 32729-1). Immunohistochemistry for vimentin identified remnants of the endothelium (arrow) of a maternal artery.

**Figure 8:**
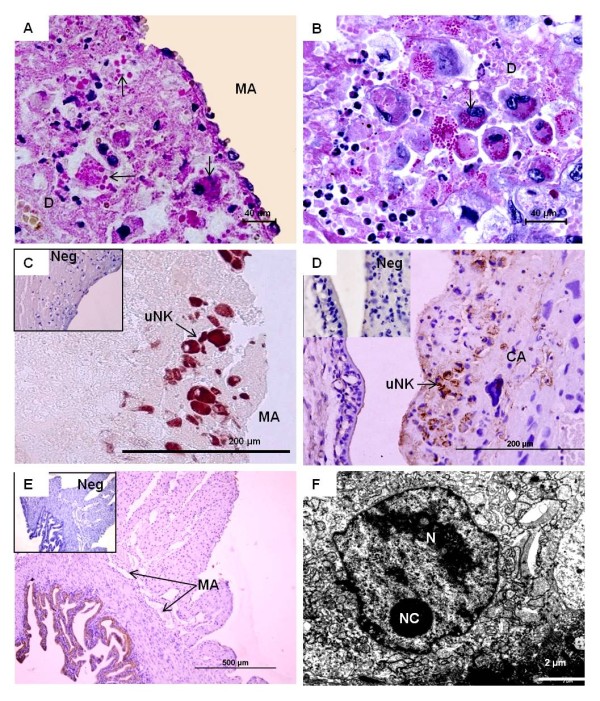
**Uterine natural killer (uNK) cells**. **(A/B) ***Oligoryzomys *in mid gestation (MZUSP 31167). PAS-positive cells accumulated near the maternal blood vessel (MA) in the decidua (D). Some were binucleated (arrows). **(C) ***Oligoryzomys *in early pregnancy (MZUSP 32735). Positive response to vimentin indicated that the cells were of mesenchymal origin, representing maternal uNK cells, not trophoblast. **(D) ***Necromys *in early pregnancy (MAV/CEMAS 04). Vimentin. In early gestation, the uNK cells were frequent also near to the outer margin of the placental disk (CA), which consisted of spongiotrophoblast and was vimentin-negative. **(E) ***Necromys *in early pregnancy (MAV/CEMAS 04). Immunostaining for cytokeratin. The negative response indicated that the invasion of trophoblast cells was limited to areas near the placenta. **(F) ***Euryoryzomys *in early pregnancy (MAV/SJB 01). TEM. The uNK cells in the decidua possessed large spherical nuclei (N) with irregular shape and distinct nucleoli (NC).

**Figure 9:**
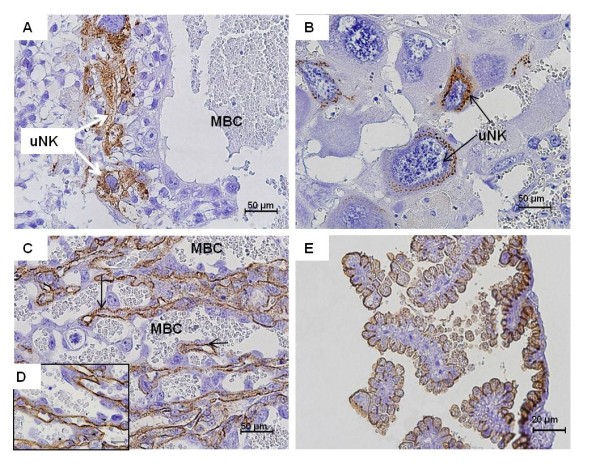
**DBA-lectin staining in *Cerradomys *in mid gestation (MZUSP/APC 1177)**. **(A) **Positively reacting cells in the decidua that were usually in close association with maternal blood vessels. **(B) **They possessed large amounts of granules and represented mature uNK cells. **(C) **DBA-lectin staining also marked cells inside the labyrinth, particularly the endothelium of the fetal capillaries (arrow). **(D) **The same at higher magnification. **(E) **The visceral yolk sac epithelium was stained by DBA-lectin.

The uterine spiral arteries entered the placenta in the decidual region (Figure 7A). As indicated by immunohistochemistry for vimentin, the mesometrial arteries possessed an intact endothelium (Figure 7B). In contrast, the endothelium of the maternal vessels near the placental disk was not completely intact. Remnants of the endothelium were detected by using vimentin staining (Figure 7C). These findings suggested that the process of trophoblast invasion was restricted to regions adjacent to the placenta. Closely associated with the maternal arteries of the decidua in all species examined, there was a large amount of both smaller and larger cells that possessed PAS-positive granules (Figures 8A,B). Such cells were often found closely grouped together. In some of these cells more than one nucleus was present (Figure 8B). In addition to PAS, these cells reacted positively for vimentin (Figure 8C), indicating that they were derived from mesenchymal tissue and thus must be of maternal origin. They were identified as uterine natural killer cells (uNK cells). In early pregnancy such PAS-positive and vimentin-positive uNK cells were also found near the developing placental disk (Figure 8D). Cells associated with the maternal arteries were not cytokeratin-positive (Figure 8E). The data once more supported their identification as uNK cells and indicated the restricted mode of trophoblast invasion into the decidua. In detail, the uNK cells mostly had large nuclei with irregular or spherical shape and some glycogen granules (Figure 8F). Using DBA-lectin staining (a phenotypic marker for uNK cells), we were able to detect positively reacting uNK cells associated with the blood vessels and inside the decidua (Figure 9A,B). However, DBA-lectin staining in sigmodontine rodents was not specific to uNK cells, marking also cells inside the labyrinth, the endothelium of the fetal capillaries in particular (Figures 9C,D), as well as the visceral yolk sac epithelium (Figure 9E).

### The visceral and parietal yolk sac

**Figure 10:**
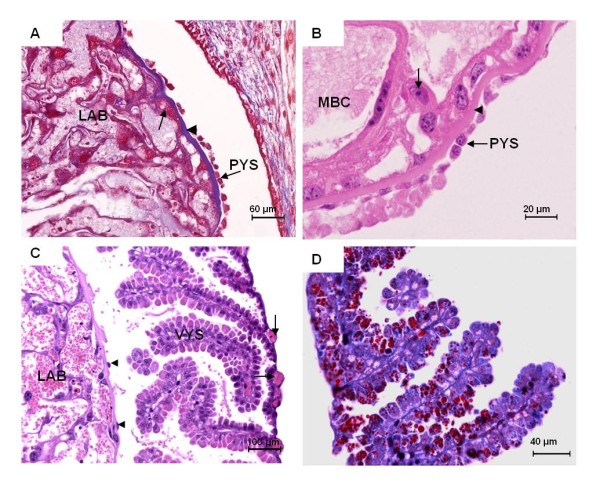
**Parietal and visceral yolk sac**. **(A) ***Necromys *in early pregnancy (MAV/CEMAS 04). Masson's Trichrome. The parietal yolk sac (PYS) was one layered and associated with a well-developed Reichert's membrane (arrowhead). **(B) ***Necromys *in early pregnancy (MAV/CEMAS 05). HE. Adjacent to the labyrinth and its maternal blood channels (MBC), trophoblast giant cells (arrow) were present. **(C) ***Cerradomys *in mid gestation (MZUSP/APC 1177-2). HE. The visceral yolk sac (VYS) was villous and supplied by vitelline blood vessels (arrows). It was close to the placental disc and the very thin parietal yolk sac that covered it (arrowheads). **(D) ***Cerradomys *in mid gestation (MZUSP/APC 1177-2). Masson's Trichrome. Positively reacting endodermal cells that were partly binucleate.

**Figure 11:**
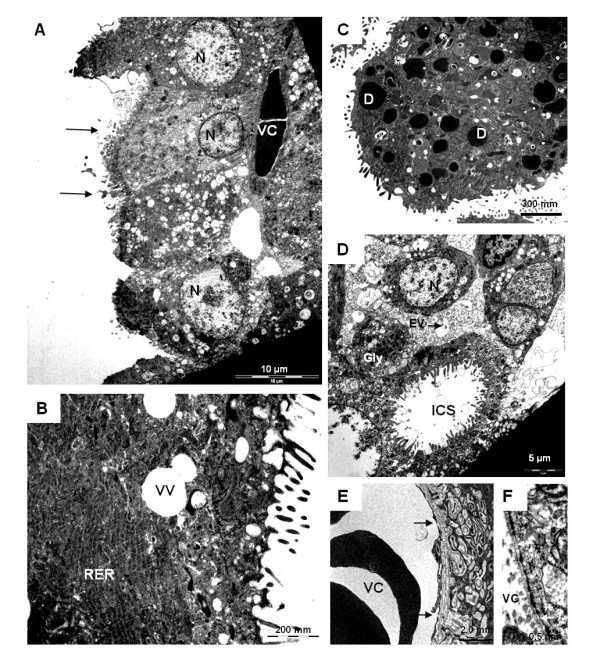
**Ultrastructure of the visceral yolk sac in mid gestation**. **(A) ***Euryoryzomys *(MAV/SJB 02). The endoderm possessed apical microvilli (arrows) and basal nuclei (N). They were close to the vitelline capillaries (VC). **(B) ***Necromys *(MZUSP/APC 1246-3). Rough endoplasmic reticulum (RER) and vacuole-like vesicles (VV) in the cytoplasm. **(C) ***Necromys *(MZUSP/APC 1246-3). Electron-dense inclusions (D). **(D) ***Euryoryzomys *(MAV/SJB 02). Glycogen deposits (Gly), endocytic vesicles (EV) and large intracellular spaces (ICS) were evident in the apical cytoplasm. **(E) ***Necromys *(MZUSP/APC 1246-3). An endothelial cell of a vitelline capillary with fenestrated regions (arrows). **(F) ***Necromys *(MZUSP/APC 1246-3). Higher magnification.

An inverted yolk sac placenta was present throughout pregnancy. It was closely attached to the labyrinthine region of the chorioallantoic placenta. The mostly one-layered parietal yolk sac covering was associated with a well-developed Reichert's membrane (Figures 10A-C). Especially in early to mid gestation the visceral yolk sac was highly villous (Figures 10C,D). The outer layer of the yolk sac consisted of visceral endoderm cells with cuboidal shape. The yolk sac endoderm cells reacted positively to PAS, including plenty of granular vesicles. Masson's Trichrome staining in *Cerradomys *suggested hemophagous activity (Figure 10D). In all species investigated, the cells of the yolk sac endoderm were separated by a basement membrane from the underlying mesoderm, which was well vascularized by vitelline blood vessels (Figures 10C, 11A). The nuclei of the visceral yolk sac endoderm were situated near the base of the cells. Their apical surface possessed numerous microvilli (Figure 11A,B). No coated pits were found. The cytoplasm included many rough endoplasmic reticulum cisterns, some glycogen granules, and vesicle-like vacuoles as well as scattered dense droplets with a great variability of shape and size (Figures 11B-D). In addition, intracellular spaces were evident in the endodermal cells (Figure 11D). The vitelline vessels included capillaries with fenestrated regions of endothelium (Figures 11E,F).

## Discussion

Sigmodontinae is a South American radiation of cricetid rodents. Placentation in this speciose subfamily has been little studied and information is limited to a single species *Calomys callosus *[17-22]. Our aim therefore was to study a broader sample. Whilst we were able to study different gestational stages from five genera these were collected mainly in the wild. In contrast to studies of laboratory rodents we did not have carefully spaced specimens of known gestational age.

As in other murid and cricetid rodents, the chorioallantoic placenta consisted of three readily identifiable zones: labyrinth, junctional zone and decidua. An inverted choriovitelline or yolk sac placenta persisted until term. Because this basic design resembles that of the mouse, for which placental development and cell lineages are best understood [2,3,5-8,32-38], our findings are discussed in relation to this species as well as to other cricetid rodents such as the golden hamster, lemming and deer mouse [13-16].

### Labyrinth and interhemal barrier

The labyrinth consisted of maternal blood channels enclosed by trophoblast and running roughly parallel to fetal capillaries. As in other murid [16] and cricetid [11,12,15,16,21] rodents, there were three layers of trophoblast, the inner two syncytial and the outer one cellular. Details of the structure of the interhemal barrier, such as the variation in thickness and the complex infolding of layer TII, were similar to what has been described in other subfamilies of cricetid rodents [12,17]. In the mouse all three trophoblast layers are derived from the same lineage [6]. They have distinct patterns of gene expression, however, and form discrete populations early in development [34]. Whilst we did find giant cells in the labyrinth, it remains to be shown if they are equivalent to the sinusoidal giant cells identified in the mouse on the basis of gene expression and polyploidy [34,37]. Although a hemotrichorial placenta is present in all murid and cricetid rodents so far examined, there are seven known variants of the interhemal barrier in rodents [39] and our previous analysis suggested that the hemotrichorial type of barrier is a derived character state [39].

### Junctional zone

As in other murid and cricetid placentae [6,13,33], there was a prominent junctional zone devoid of fetal vessels but with large trophoblast-lined maternal blood spaces. Two distinct types of trophoblast are found here: spongiotrophoblasts and glycogen cells. They were long thought to have a common origin but glycogen cells may be derived from precursors in the ectoplacental cone that, like them, express protocadherin 12 [40]. In the mouse placenta at E16.5 about 40% of the spongy zone is made up of glycogen cells but the proportion diminishes towards term [3]. We found relatively few glycogen cells in our specimens but this was difficult to interpret since gestational ages were not known.

### Trophoblast giant cells

The classical giant cells of the rodent placenta, first recognized by their large size and high degree of polyploidy [41], are now referred to as parietal TGCs [34]. A few of them, sometimes known as primary giant cells, are derived from the mural trophectoderm, but the majority comes from the *Tpbpa*-negative lineage of the polar trophectoderm [6]. In the mouse they form a nearly continuous layer that marks the boundary between fetal and maternal tissues, although this boundary is breeched once the glycogen cells begin to invade the decidua. As expected, parietal TGCs were found at this location in the sigmodont placenta. In addition, in three genera, the giant cells formed a layer many cells thick at the margin of the disk. A similar accumulation of giant cells is not known from mouse placenta where the total population of parietal TGCs is estimated to be only twenty thousand [3].

### Decidua and maternal blood vessels

The extent to which trophoblast invades maternal blood vessels differs among murid rodents [9]. In the mouse it has been thought for many years that trophoblast invasion is confined to vessels in the fetal part of the placenta [42]. In contrast, in the rat, trophoblast invasion by the endovascular route plays an important part in remodeling of the uterine spiral arteries [9,43]. More recently it has been recognized that in mouse some trophoblasts migrate by a perivascular route and end up in the lumen of the maternal arteries [2,34]. Trophoblast invasion of maternal arteries certainly occurs in the golden hamster, a cricetid rodent [13,44]. In contrast, in sigmodonts, we did not find cytokeratin-positive cells in the walls of the spiral arteries and the vessel endothelium was largely intact. Cytokeratin-positive cells were not found in the mesometrial triangle. Whilst this is suggestive of shallow trophoblast invasion in sigmodonts, it clearly is an aspect that needs to be systematically explored in specimens of known gestational age.

### Uterine NK cells

The uNK cells are the dominant leukocyte population in the gravid uterus of rodents and primates. In human pregnancy they are responsible for the earliest events in spiral artery transformation, which occur prior to trophoblast invasion [45]. In the mouse they play an even greater role in adaptation of the maternal arteries, which fail to widen in the absence of uNK cells [46]. Although murine uNK cells produce a number of cytokines, the key molecule appears to be interferon-gamma [47]. For cricetid rodents, the presence of uNK cells in the walls of transformed spiral arteries was first shown in the golden hamster [13]. We have shown that they are present in large numbers in sigmodont rodents and are similarly associated with the spiral arteries. It is likely that they play an important role in vessel widening and remodeling similar to what has been shown experimentally in the mouse.

### Parietal and visceral yolk sac

The yolk sac was no different in structure from what has been described for other murid and cricetid rodents [10,14,48-50]. Recently it was reported [50] that there are no caveolae-like structures in the yolk sac endoderm of the mouse. Likewise, this appears to be the case in *Necromys *and *Euryoryzomys*, the two sigmodonts we examined by TEM. This is an interesting contrast to what consistently is found in the guinea pig [51] and other hystricognath rodents [e.g. 31,52,53].

### Implications for the biology of sigmodont rodents

The sigmodont rodents reached South America at the time of the Great American Interchange in the Pliocene Epoch or perhaps even earlier [54]. They underwent a rapid radiation that led them to occupy a variety of habitats, including the xeric biomes *Cerrado *and *Caatinga *of Brazil [54,55]. Despite their potential importance as bioindicators [28] and their undoubted significance as reservoirs of disease [27], they are much less well studied than, for example, the hystricomorph rodents of Latin America (e.g. [30,31,51-53,56-58]).

In most respects placentation in this subfamily closely resembles what has been described for other cricetid rodents [13,15]; the close similarity in the fine structure of the interhemal barrier has already been remarked upon. As we have shown elsewhere, the hemotrichorial type of placenta first appeared in the common ancestor or murid and cricetid rodents [39]. Likewise the prominent role of maternal uNK cells in placentation is a feature that sigmodonts share with other cricetid and murid rodents [13,46]. The most striking finding in the present material was the relative abundance of trophoblast giant cells first described in *Calomys *[19] and here extended to a further five genera. In murid rodents giant cells produce a wide range of hormones and cytokines such as proliferin [59] and the significance of the expanded giant cell population in sigmodonts cries for closer attention.

It is, however, unlikely that our understanding of placentation in this subfamily can be further advanced by field studies. It would be better to establish a breeding program and obtain a series of time dated pregnancies from a single species such as *Calomys callosus *or *Necromys lasiurus*, both of which have been bred in captivity. The feasibility of such an approach is apparent from an earlier study of trophoblast invasion at the start of pregnancy [17].

## Conclusions

In summary the general aspect of the fetal membranes in Sigmodontinae resembled that found in other cricetid rodents. The chorioallantoic placenta was organized in a labyrinthine zone, junctional zone and decidua and an inverted yolk sac persisted until term. The interhemal barrier was of the hemotrichorial type. The junctional zone was comprised of spongiotrophoblast, glycogen cells and trophoblast giant cells. Compared to murid rodents there were much larger numbers of giant cells and in some genera these were seen to congregate at the periphery of the placental disk. Glycogen cells were found to invade the decidua but we did not identify trophoblast in the walls of the deeper decidual arteries. In contrast these vessels were surrounded by large numbers of uNK cells. This survey of wild-trapped specimens from five genera is a useful starting point for the study of placentation in an important subfamily of South American rodents. We note, however, that some of these rodents can be captive bred and recommend that future studies focus on the study of time dated pregnancies.

## Competing interests

The authors declare that they have no competing interests.

## Authors' contributions

AMC and MAM devised the study and participated in its design and coordination. POF performed the major part of the histological analysis. CEA, ACM, MFO and AMM participated in the study design and analysis. AMC, AMM and POF wrote the manuscript. All authors read and approved the final manuscript.
